# Accelerated degradation testing impacts the degradation processes in 3D printed amorphous PLLA

**DOI:** 10.3389/fbioe.2024.1419654

**Published:** 2024-07-05

**Authors:** Luke P. Malone, Serena M. Best, Ruth E. Cameron

**Affiliations:** Department of Materials Science and Metallurgy, Cambridge Centre for Medical Materials, University of Cambridge, Cambridge, United Kingdom

**Keywords:** poly-l-lactic acid, degradation, biodegradable polymers, crystallinity, molecular weight, 3D printing, electrospinning

## Abstract

Additive manufacturing and electrospinning are widely used to create degradable biomedical components. This work presents important new data showing that the temperature used in accelerated tests has a significant impact on the degradation process in amorphous 3D printed poly-l-lactic acid (PLLA) fibres. Samples (c. 100 μ m diameter) were degraded in a fluid environment at ${37\;}^{{}^{\circ}}$ C, ${50\;}^{{}^{\circ}}$ C and ${80\;}^{{}^{\circ}}$ C over a period of 6 months. Our findings suggest that across all three fluid temperatures, the fibres underwent bulk homogeneous degradation. A three-stage degradation process was identified by measuring changes in fluid pH, PLLA fibre mass, molecular weight and polydispersity index. At ${37\;}^{{}^{\circ}}$ C, the fibres remained amorphous but, at elevated temperatures, the PLLA crystallised. A short-term hydration study revealed a reduction in glass transition (Tg), allowing the fibres to crystallise, even at temperatures below the dry Tg. The findings suggest that degradation testing of amorphous PLLA fibres at elevated temperatures changes the degradation pathway which, in turn, affects the sample crystallinity and microstructure. The implication is that, although higher temperatures might be suitable for testing bulk material, predictive testing of the degradation of amorphous PLLA fibres (such as those produced via 3D printing or electrospinning) should be conducted at ${37\;}^{{}^{\circ}}$ C.

## 1 Introduction

Additive manufacturing has the potential to revolutionise medical device fabrication. The range of potential applications is considerable, encompassing everything from drug delivery devices (Bácskay et al., 2022), to stents (Guerra et al., 2018), screws (MacLeod et al., 2020), meshes (Ren et al., 2022) and 3D printed organs (George et al., 2023). Much research focuses on the concept of personalised device fabrication, producing devices that perfectly match the individual patient requirements (Nagarajan et al., 2018). This in turn could pave the way to a reduction in recovery time, material wastage or device cost (Goyanes et al., 2015; Qamar et al., 2019). Similarly, electrospinning has been used to fabricate scaffolds for a variety of biomedical applications including cartilage repair (Castilho et al., 2019), bone tissue regeneration (Lee et al., 2013) and tendons or ligaments (Hochleitner et al., 2018). The deposited scaffold architectures are highly controllable, forming structures that can mimic the native tissue environment (Kai et al., 2014).

Poly-L-lactic acid (PLLA) is an example of a widely used man-made poly ( α -hydroxy ester). As a material, PLLA is commonplace within 3D printing and electrospinning, which combined with its good biocompatibility (Wang et al., 2017), has led to it being suggested for a range of biomedical applications (Tamai et al., 2000; Song et al., 2017; Yoon et al., 2017; Singh et al., 2019; Zhao et al., 2020; Hu et al., 2021; Jumat et al., 2021). Crucial to its success is the ability to tailor the deposited microstructure to the target application. Roper et al. have shown that the chain alignment of PLLA can be controlled during the 3D printing of thin (100 μ m) single layer lines (Roper et al., 2022). The resulting microstructure was found to be aligned but amorphous. These lines could be utilised in the manufacture of thin polymeric struts, with the deposited thickness matching the requirements for certain mesh applications (de Tayrac et al., 2008) or within blood flowing areas (McCormick et al., 2018), where previous thick PLLA structures have resulted in long term thrombosis issues (Ali et al., 2018; Sakamoto et al., 2018).

One of the major advantages of PLLA as a biomedical material is its ability to degrade *in-vivo* in a controlled manner over a long time frame. The degradation produces harmless by-products that can be processed naturally via the Cori cycle (da Silva et al., 2018). However, this specific advantage (its long degradation time), is also a major drawback for studying the material within a research setting. PLLA requires several years to completely degrade within the body or similar *in vitro* conditions. As a result, many researchers use accelerated degradation testing to speed up the process. Accelerated degradation testing of poly ( α -hydroxy esters) can take multiple forms but often involves either elevated temperatures (Weir et al., 2004a) or harsh chemical environments (Vasanthan and Ly, 2009; Chausse et al., 2023). When elevated temperatures are applied, the Arrhenius equation is used to extrapolate to body temperature. The elevated temperatures can be divided into one of two categories: above the starting glass transition temperature (Tg) of the dry material or below. However, the validity and applicability of either category to predict the *in-vivo* lifespan require further investigation (Agrawal et al., 1997). When Weir et al. investigated the degradation behaviour of 0.8 mm thick annealed PLLA they raised concerns regarding the validity of accelerated degradation testing when the temperature was raised above the starting Tg of the dry material (Weir et al., 2004a). However, a study by Bergsma et al. involving 2 mm thick semi-crystalline PLLA concluded that temperatures above the starting Tg were suitable for predicting the *in-vivo* lifespan and degradation pathway (Bergsma et al., 1995). Additionally, fluid immersion has been shown to effect the Tg of the polymer (Vyavahare et al., 2014). Uncertainties persist regarding the validity of accelerated degradation testing to predict *in-vivo* lifespan and the temperatures that are appropriate to use.

The degradation pathway of PLLA is commonly characterised as a bulk degradation process, with the diffusion of fluid into the structure faster than the degradation rate of the polymer backbone (Pierre and Chiellini, 1987; Deb and Di Silvio, 2009; von Burkersroda et al., 2002). Initially, water or fluid is absorbed into the material. Several studies have suggested that within a semi-crystalline microstructure, the amorphous regions are selectively degraded first (Fischer et al., 1973; Li et al., 1990; Pistner et al., 1993; Tsuji and Ikada, 1997; Tsuji and Ikada, 1998; Tsuji et al., 2000; Chye et al., 2005), with the eventual degradation of crystalline domains shown to proceed from the edges inwards (Fischer et al., 1973).

The impact of manufacturing technique on the degradation rate needs to be carefully considered (Vaid et al., 2021). Chausse et al. previously investigated the degradation of 3D printed PLLA stents (starting strut thickness 120.9 μ m) manufactured using solvent-cast direct-writing at ${50\;}^{{}^{\circ}}$ C. They concluded that the stents underwent a bulk degradation process (Chausse et al., 2023). However, all the stents were thermally treated at ${80\;}^{{}^{\circ}}$ C for 12 h before degradation testing, with the starting microstructure semi-crystalline. There is no agreed consensus regarding the influence of starting crystallinity on the degradation rate or behaviour, or the validity of extrapolating from a semi-crystalline microstructure to an amorphous microstructure (Li et al., 1990; Pistner et al., 1993; Duek et al., 1999; Tsuji et al., 2000; Pantani and Sorrentino, 2013). Therefore, for an starting amorphous microstructure, independent degradation testing at ${37\;}^{{}^{\circ}}$ C needs to be conducted.

Following the initial degradation of the polymer chain backbone, the diffusion of degradation products out of the structure is slow. Only when the molecular weight of the formed fragments and the surrounding material are sufficiently small, can they diffuse out of the structure, marking the onset of mass loss and pH change (Göpferich, 1997). In the time between the initial polymer chain degradation, but prior to the diffusion of the molecular weight fragments out of the structure, the degradation products can catalyse the reaction via autocatalysis (leading to heterogeneous bulk degradation) (Vert et al., 1991). However, autocatalysis has not been reported in all degradation studies and is strongly linked to the material starting thickness and the product’s diffusion path to the sample exterior. Multiple studies have found that with an increase in sample thickness, the degradation rate increases (Grizzi et al., 1995; Lu et al., 1999; Witt and Kissel, 2001). With an increase in sample thickness, the diffusion path for the release of degradation products is greater, and their build-up can then autocatalyse the reaction (Fu et al., 2000). However, the critical thickness at which autocatalysis becomes significant is highly dependent upon the starting polymer and manufacturing technique. Vaid et al. reported no evidence of autocatalysis in PLA fibres (starting material contained less than 2% D-isomer) 10–15 μ m in diameter (Vaid et al., 2021). They suggested that the lack of a bulk erosion within their samples was due to their high starting crystallinity from the fibre-forming process and emphasised the importance of considering the starting microstructure when considering degradation behaviour. However, Li et al. reported the presence of autocatalysis for PLLA degraded at ${37\;}^{{}^{\circ}}$ C over 2 years for both a starting amorphous microstructure and a semi-crystalline microstructure (Li et al., 1990). In both materials, the starting thickness was 2 mm, significantly greater than Vaid et al. study. There is no clear consensus regarding a critical thickness for heterogeneous degradation of PLLA or the effect of materials processing history and starting microstructure on the subsequent degradation behaviour. A study needs to investigate whether autocatalysis occurs within amorphous material, with thickness relevant to future mesh applications.

This study aims to investigate how 100 μ m thick 3D printed amorphous PLLA fibres degrade at ${37\;}^{{}^{\circ}}$ C. The research focuses on the changes that occur within the microstructure over 6 months. Additionally, this study aims to investigate the validity of accelerated temperature degradation and extrapolation back to ${37\;}^{{}^{\circ}}$ C. Three different temperatures are investigated: body temperature ( ${37\;}^{{}^{\circ}}$ C), an elevated temperature below the Tg of the dry material ( ${50\;}^{{}^{\circ}}$ C), and an elevated temperature above the dry Tg ( ${80\;}^{{}^{\circ}}$ C). Various tests including optical microscopy, X-ray diffraction (XRD), gel permeation chromatography (GPC), mechanical testing and differential scanning calorimetry (DSC) are used to characterise the resulting microstructure. Based on the new data, an approach for future PLLA degradation testing involving 3D printed amorphous fibres, with a starting thickness suitable for biodegradable stents and meshes, is suggested.

## 2 Materials and methods

### 2.1 Materials

PLLA (2500HP) NatureWorks pellets were extruded using a Noztek extruder at $182^{\;{}^{\circ}}$ C, 1,200 rpm with a 1.75 mm nozzle. Prior to printing, all extruded filament was vacuum dried for 24 h. A Prusa MK3s + Fused Filament Fabrication (FFF) printer, with a mounted Titan Extruder, was used. The Titan Extruder increased the gear ratio 3:1. The print bed was preheated to ${55\;}^{{}^{\circ}}$ C and the nozzle was preheated to $215^{\;{}^{\circ}}$ C. Samples were extruded from a 400 μ m nozzle diameter, at a 3,000 mm/min print speed. A 200 μ m PVA sacrificial support layer was used during printing, as defined in previous work (Roper et al., 2021). This printing setup created highly orientated but amorphous microstructures (Roper et al., 2022). For each sample approximately 10 PLLA lines 10 cm in length, approximately 100 μ m thick and 450 μ m wide were printed. An example of the starting cross section from samples produced under similar conditions can be seen in our previous work (Roper et al., 2021). The fibres were printed in the *x* direction with a final support line printed in the *y* direction to maintain line separation throughout the degradation. These support lines ensured that all fibres degraded homogeneously and were dried fully prior to final mass measurement.

### 2.2 Hydration study

To investigate the effect of fluid immersion on the glass transition, a short hydration study involving the as printed PLLA was conducted. Samples were immersed for 3 days in fluid at either ${37\;}^{{}^{\circ}}$ C or ${50\;}^{{}^{\circ}}$ C. After 3 days, a section of the sample was immediately placed into the DSC (while still hydrated). The remainder of the sample was vacuum dried for 3 days and then placed in the DSC. The DSC was run from 0 to $210^{\;{}^{\circ}}$ C, whilst under a 50 mL/min nitrogen flow rate, at a $10^{\;{}^{\circ}}$ C/min heating rate.

### 2.3 Degradation testing

After printing, the PVA was peeled off. Samples were then weighed and split into groups based on their degradation time and temperature. There were three samples for each degradation time and temperature, with the total starting group mass approximately the same. Samples were placed into 7 mL individual bijou tubes with phosphate buffer saline (PBS) pipetted on top in a sample mass to fluid ratio of 1 mg:125 mL. The bijou tubes were placed into one of three temperature-controlled environments ( ${37\;}^{{}^{\circ}}$ C, ${50\;}^{{}^{\circ}}$ C or ${80\;}^{{}^{\circ}}$ C). For PLLA degraded at ${37\;}^{{}^{\circ}}$ C or ${50\;}^{{}^{\circ}}$ C, samples were removed at days 15, 30, 50, 70, 100, 120 and 180. For PLLA degraded at ${80\;}^{{}^{\circ}}$ C, samples were removed at 30 min, 1 h, 2 h and 1, 2, 4, 7, 10, 14 and 22 days, due to the faster degradation at this temperature.

After the allocated degradation time, samples were filtered, washed twice in deionised water and vacuum dried for 3 days.

### 2.4 Sample characterisation

#### 2.4.1 pH change

Following degradation and removal from the temperature controlled environment, the fluid pH was measured three times. The error in the pH measurement was determined by the resolution of the probe.

#### 2.4.2 Mass loss

After 3 days of vacuum drying, samples were weighed three times to find their final dry mass. The percentage mass loss during degradation was calculated using Eq. (1).

$$M_{L}=\frac{M_{f}-M_{s}}{M_{s}}*100$$ (1)

where $M_{L}$ is the percentage mass lost, $M_{f}$ final dry sample mass and $M_{s}$ start mass.

The percentage error for each time and temperature was calculated by taking the standard deviation in mass loss for each sample and then using standard error propagation rules to combine all samples of the same degradation time and temperature.

#### 2.4.3 Molecular weight

Gel-permeation chromatography (GPC) was conducted on a 1,260 Infinity II Multi-Detector GPC/SEC System at the University of Manchester, supported by the Henry Royce Institute for Advanced Materials. 10–15 mg of a dried sample was dissolved in chloroform (3 mg/mL). The dissolved samples were filtered through a 0.2 μ m syringe prior to insertion into the machine. The flow volume was 100 μ L, and a 1 mL/min flow rate was used. A polystyrene standard was used for calibration. Analysis was conducted using Aglient GPC/SEC software. GPC was conducted to find the number average molecular weight (Mn), weight average molecular weight and the polydispersity index (PDI). The GPC was run in three separate batches. Each time a batch was run, a NatureWorks PLLA pellet was run first. The spread in reported Mn values for the pellet generated a conservative 6.0% error in each reported Mn value and 8.3% error in the reported PDI value.

#### 2.4.4 X-ray diffraction

Wide-angle X-ray scattering (WAXS) scans on dried samples were undertaken using a Bruker D8 Advance diffractometer with a Cu-K α source ( λ = 1.54059 *Å*). To quantify the change in crystallinity, a Rietveld refinement was carried out using Topas software V7.21. The Rietveld refinement used a least-squares fitting approach to map experimental data to a peak list. The peak list was generated using a computational structural model. A z-matrix for all 90 carbon, oxygen and hydrogen atoms, 89 bond lengths, 88 bond angles and 87 torsional angles was used as an input for the axisymmetric polymer unit. The data set was built using the work of Wasanusuk et al. (Wasanasuk et al., 2011), assuming an orthorhombic unit cell. The bond lengths and angles were fixed, but torsional angles were able to rotate. A penalty and anti-bump penalty was introduced to stop large atomic deviation from the original coordinate site. A dummy variable linked the end of one unit cell to the next. Finally, a preferred chain orientation along the (001) direction was taken into account. The amorphous background was modelled using a Chebyshev function, and crystalline peaks were assumed to be Gaussian. The Lorentz-polarisation was zero. To maximise the model fitting, the atomic coordinates and unit cell size could change. Various space groups were initially modelled (P $12_{1}$ 1, P $2_{1}$ 11 and ${P2}_{1}2_{1}2_{1}$ ) all assuming a 10/3 unit packing. However, it was determined that ${P2}_{1}2_{1}2_{1}$ had the best fitting and was used for all analysis.

To validate the computational model, PLLA was mixed with the diffraction standard corundum in three different ratios. The corundum was supplied by MetPrep (Alpha 1.0 micron category number 159980). Across all three ratios, the model was able to correctly predict the final corundum to polymer ratio within ± 10%. Based on the error in the final corundum fitting a conservative ± 10% error bar is plotted on percentage crystallinity values. Following validation, the final model was fitted to each experimental WAXS data set.

#### 2.4.5 Thermal properties

Thermal properties were measured using a TA Instruments differential scanning calorimetry (DSC) 2,500. The DSC was run in batches, with all degraded material from a single fluid temperature run at the same time. Before each batch was run, a sample of as printed material was run. The initial material characterisation values and associated errors were calculated based upon the average and standard deviation of this data. For every sample, 2–5 mg of dried material was sealed in an aluminium pan, and DSC was conducted using a $10^{\;{}^{\circ}}$ C/min heating rate from 0 to $210^{\;{}^{\circ}}$ C under a 50 mL/min nitrogen flow rate. The glass transition temperature (Tg), cold crystallisation temperature, melting temperature (Tm) and enthalpy of fusion were recorded.

#### 2.4.6 Mechanical properties

Tensile mechanical testing was undertaken using a Tinius Olsen 1ST universal tensile testing machine. A 25 N load cell with self-tightening grips was used. Sample gauge length was measured and approximately 20 mm. The strain rate was 0.001 $s^{-1}$ . For each time and temperature, five individual dried sample lines were measured to failure. The data were analysed using a custom script in MATLAB. The initial ramp-up section of the curve was removed, with the Young’s modulus recorded from the steepest linear section. Yield stress and strain were measured at a 0.2% strain offset. The ultimate tensile strength (UTS) was defined as the maximum stress the sample reached before failure, with the corresponding strain recorded as the failure strain.

Sample cross section was recorded using optical microscopy in reflection mode. Samples were sectioned into three pieces. A ×10 objective lens was used to find sample width and thickness for individual lines prior to mechanical testing. The cross sectional area was approximately rectangular in shape, and the formula for area of a rectangle was used to calculate the cross sectional area (Roper et al., 2021).

## 3 Results

### 3.1 Initial material characterisation

**FIGURE 1:**
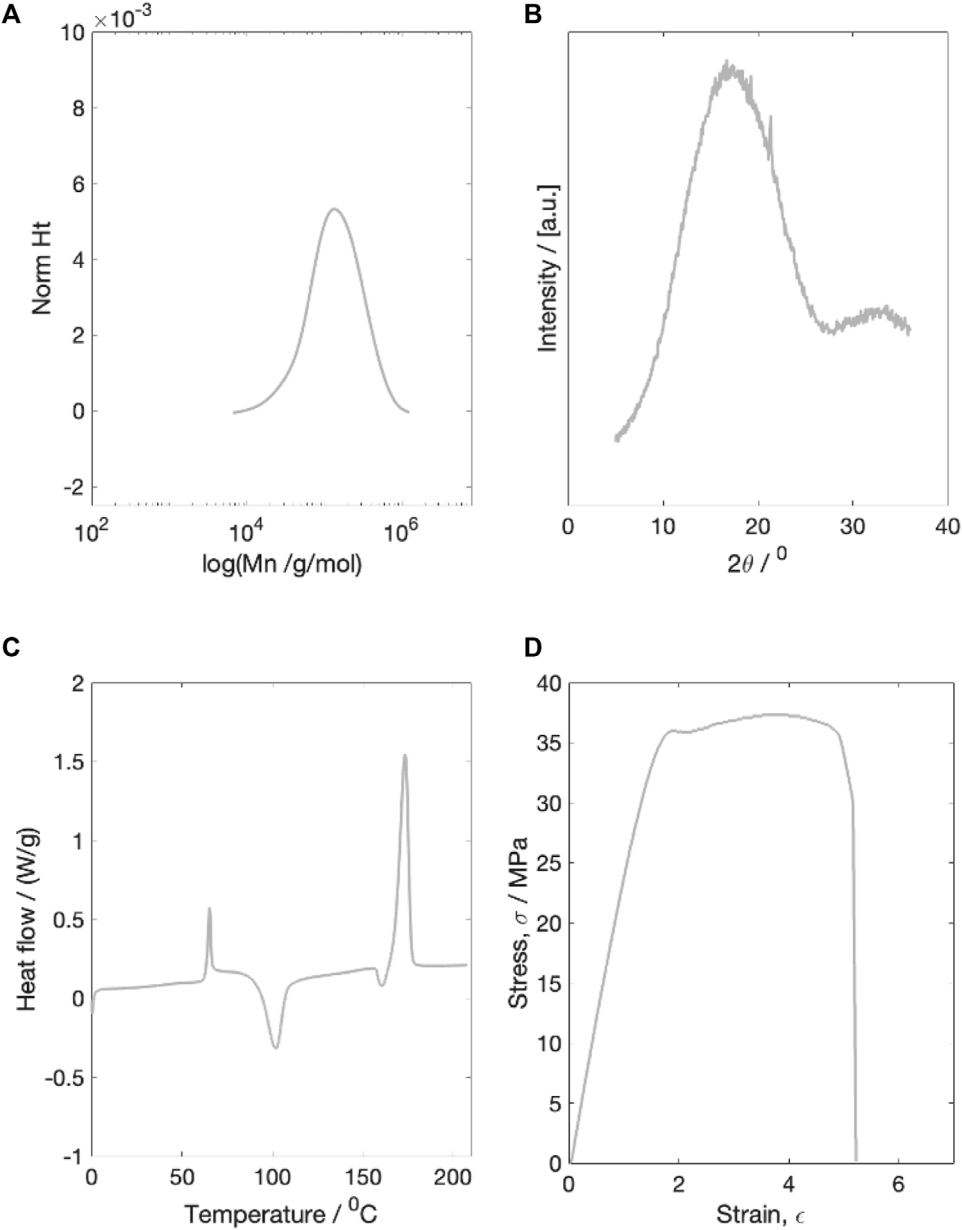
Example characterisation plots of the dried initial material. **(A)** molecular weight characterisation **(B)** X-ray diffraction characterisation **(C)** Thermal properties characterisation **(D)** Example stress-strain plot for a single dried starting sample. All four tests were run on dried material.

Prior to degradation testing, the initial molecular weight, X-ray diffraction scattering, thermal properties and mechanical properties of the as printed PLLA fibres were characterised (Figure 1).

#### 3.1.1 Molecular weight

The dry number average starting molecular weight (Mn) was measured to be 100,598 g/mol. The starting PDI was 1.79 ± 0.15. The GPC curve contained a single peak (Figure 1a).

#### 3.1.2 X-ray diffraction

The initial dry material was completely amorphous, with a single broad WAXS peak and a calculated crystallinity of 4.5 ± 10% (Figure 1B).

#### 3.1.3 Thermal properties

Thermal characterisation of the initial dry material revealed an average Tg of 61.5 ± $1.5^{\;{}^{\circ}}$ C, cold crystallisation temperature of 100.3 ± $5.1^{\;{}^{\circ}}$ C, and a single melting peak at 172.5 ± $3.2^{\;{}^{\circ}}$ C (Figure 1C). Prior to melting, a small endothermic dip was recorded.

#### 3.1.4 Mechanical properties

The average dry starting Young’s modulus was measured to be 2.00 ± 0.46 GPa. The average material yield stress and strain were 31.27 ± 3.98 MPa and 1.81 ± 0.27 respectively, before a final average failure recorded stress and strain values of 38.24 ± 4.31 MPa and 4.42 ± 1.51 respectively (Figure 1D).

### 3.2 Hydration study

**FIGURE 2:**
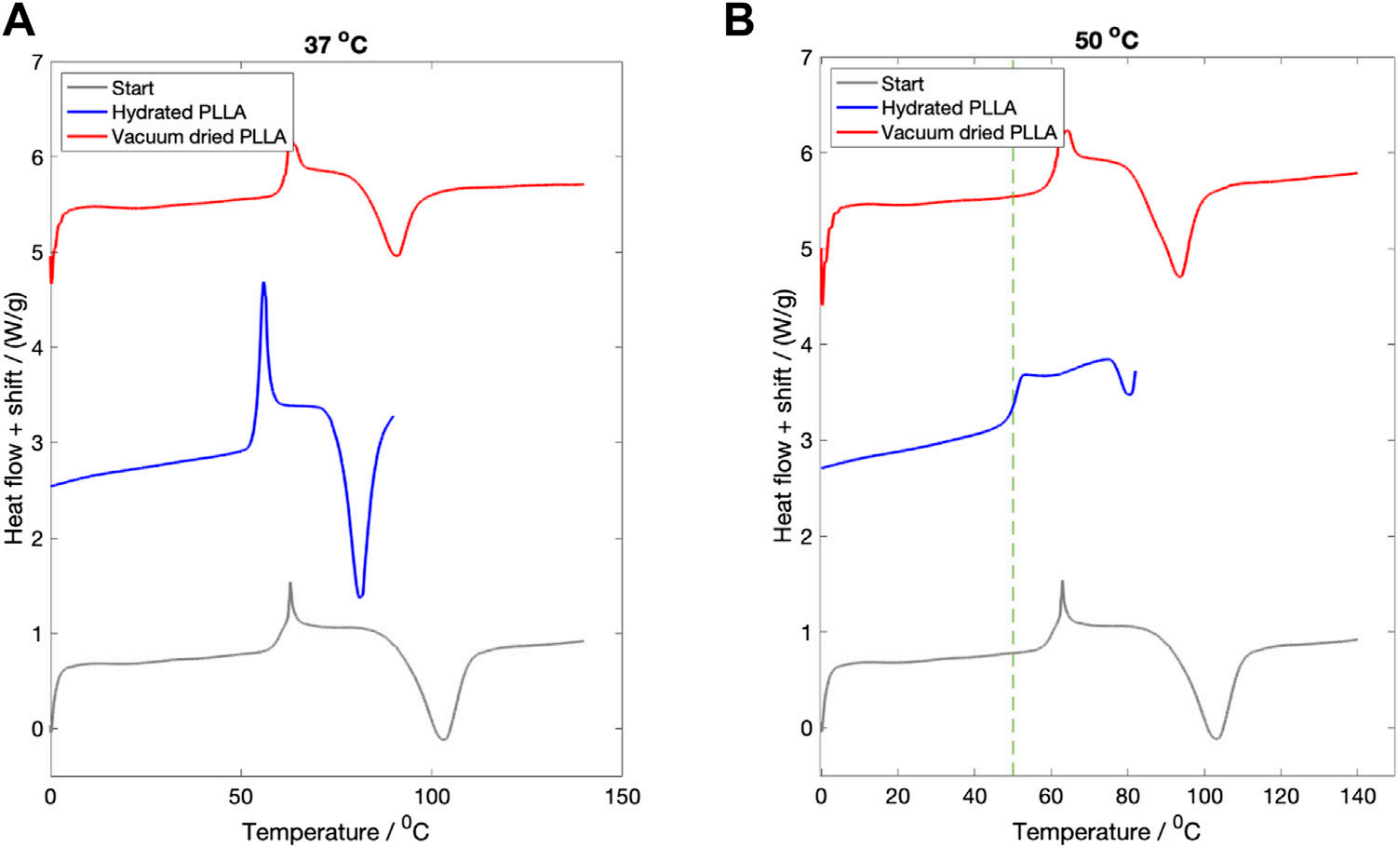
The effect of fluid immersion for 3 days on the recorded thermal properties. **(A)** Hydration at ${37\;}^{{}^{\circ}}$ C. The starting DSC curve for the dry material is shown in grey. After 3 days immersion, the measured Tg for the hydrated material was lowered slightly (blue line). After vacuum drying (red line) the Tg returned back to its start value. **(B)** Hydration at ${50\;}^{{}^{\circ}}$ C. In the hydrated state (blue line) the Tg was measured to be ${50\;}^{{}^{\circ}}$ C. Upon vacuum drying, the Tg returned to its original value (red line). In both figures a vertical shift (approximately 2.5 W/g) between each line has been introduced for clarity. In Figure **(B)** a green dotted line marking the temperature of the surrounding fluid has been added.

The DSC curves for samples hydrated in PBS for 3 days at ${37\;}^{{}^{\circ}}$ C fluid are shown in Figure 2A. The starting material curve is plotted in grey. The hydrated material (still wet) is plotted in blue and the vacuum dried material curve is plotted in red. A vertical phase shift between each of the lines has been introduced for clarity. In the hydrated state, the Tg reduced from 61.5 ± $1.5^{\;{}^{\circ}}$ C to 54.5 ± ${1.0\;}^{{}^{\circ}}$ C, before returning to its original value upon drying. Within the hydrated state, a prominent enthalpy relaxation peak can be seen. The peak height slightly reduced upon drying, possibly due to structural changes occurring during the water desorption (Vyavahare et al., 2014).

The same three DSC curves for PLLA hydrated at ${50\;}^{{}^{\circ}}$ C for 3 days are shown in Figure 2B. A boundary line marking the ${50\;}^{{}^{\circ}}$ C fluid temperature has been added. In the hydrated state (blue curve) the glass transition reduced from 61.5 ± ${1.0\;}^{{}^{\circ}}$ C to 50.6 ± ${0.5\;}^{{}^{\circ}}$ C. No enthalpy relaxation signal can be seen in the hydrated state. Upon vacuum drying, the Tg then returned to its original value.

The study was not undertaken at ${80\;}^{{}^{\circ}}$ C, given that mass loss and pH changes at this fluid temperature occurred within the first 24 h (Figure 4C).

### 3.3 Degradation study

#### 3.3.1 Degraded sample appearance

**FIGURE 3:**
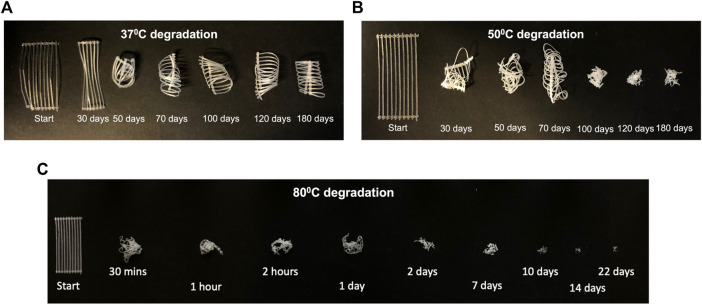
Change in sample appearance after drying for samples degraded at **(A)** ${37\;}^{{}^{\circ}}$ C, **(B)** ${50\;}^{{}^{\circ}}$ C and **(C)** ${80\;}^{{}^{\circ}}$ C. Starting samples were 10 cm in length.

The changes to sample appearance after degradation and sample drying, are shown in Figure 3. At ${37\;}^{{}^{\circ}}$ C, samples remained transparent throughout the degradation period (Figure 3A) and all samples maintained their structural integrity throughout the 180 days degradation period. The sample coiling arose due to sample placement within the bijou tube and was present prior to sample drying. Samples degraded at ${50\;}^{{}^{\circ}}$ C (Figure 3B) turned white during the first 30 days. After 100 days, upon removal from the bijou tube, samples were no longer intact. Samples degraded at ${80\;}^{{}^{\circ}}$ C (Figure 3C) turned white and began to lose their structural integrity within the first 24 h.

#### 3.3.2 Mass loss and pH change

**FIGURE 4:**
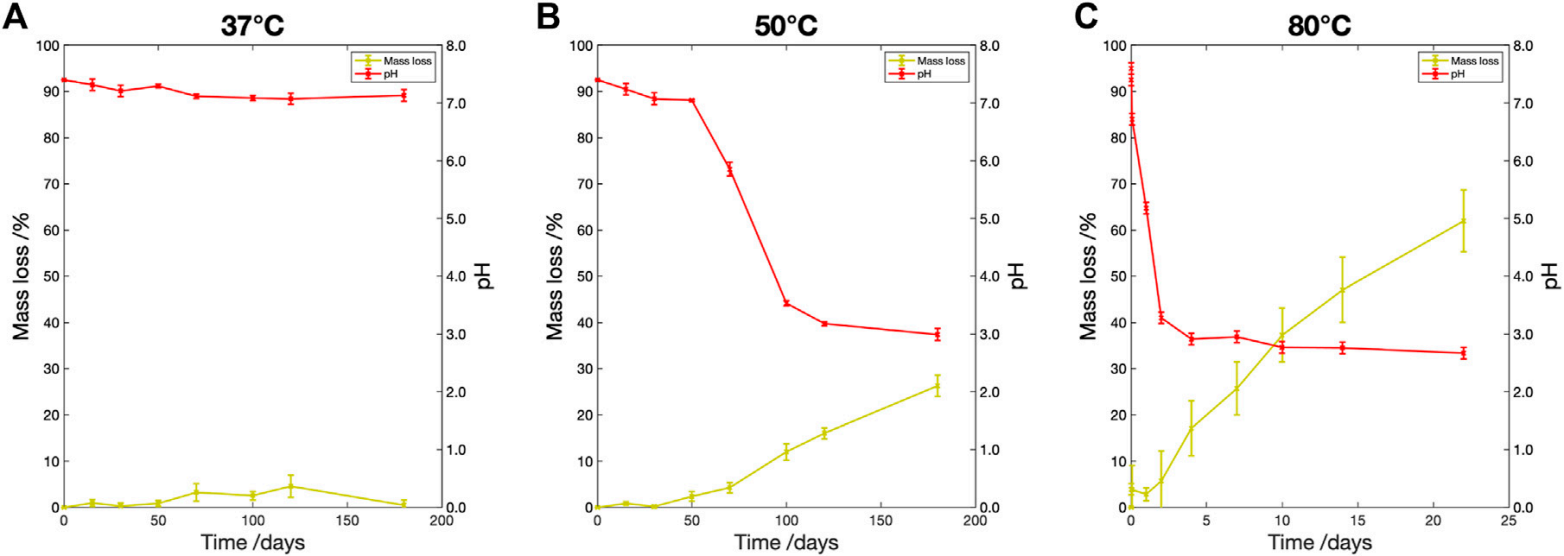
Percentage mass loss (yellow) and pH change (red) vs. degradation time for samples degraded in PBS at **(A)** ${37\;}^{{}^{\circ}}$ C, **(B)** ${50\;}^{{}^{\circ}}$ C and **(C)** ${80\;}^{{}^{\circ}}$ C. The percentage mass loss is shown on the left vertical axis, the fluid pH on the right vertical axis.

The percentage mass loss and pH change are shown in Figure 4. At ${37\;}^{{}^{\circ}}$ C (Figure 4A) the fluid maintained a constant pH and after 180 days no significant mass loss was recorded.

At ${50\;}^{{}^{\circ}}$ C (Figure 4B) during the first 50 days there was little change to fluid pH or sample mass. However, by Day 70, a 4.2 ± 1.1% mass loss was recorded and the pH reduced from 7.4 to 5.9 ± 0.1. The pH plateaued after 100 days, with the minimum recorded pH 3.0 ± 0.1. The sample continued to lose mass loss with a maximum recorded loss of 26.3 ± 2.3% after 180 days.

At ${80\;}^{{}^{\circ}}$ C (Figure 4C) rapid mass loss and pH change were recorded within the first 24 h. The pH shifted to 2.9 ± 0.1 after 4 days. There was no further change in the fluid pH after this point. The sample continued to lose mass throughout, with a maximum recorded loss of 62.0 ± 6.7% after 22 days.

#### 3.3.3 Molecular weight

**FIGURE 5:**
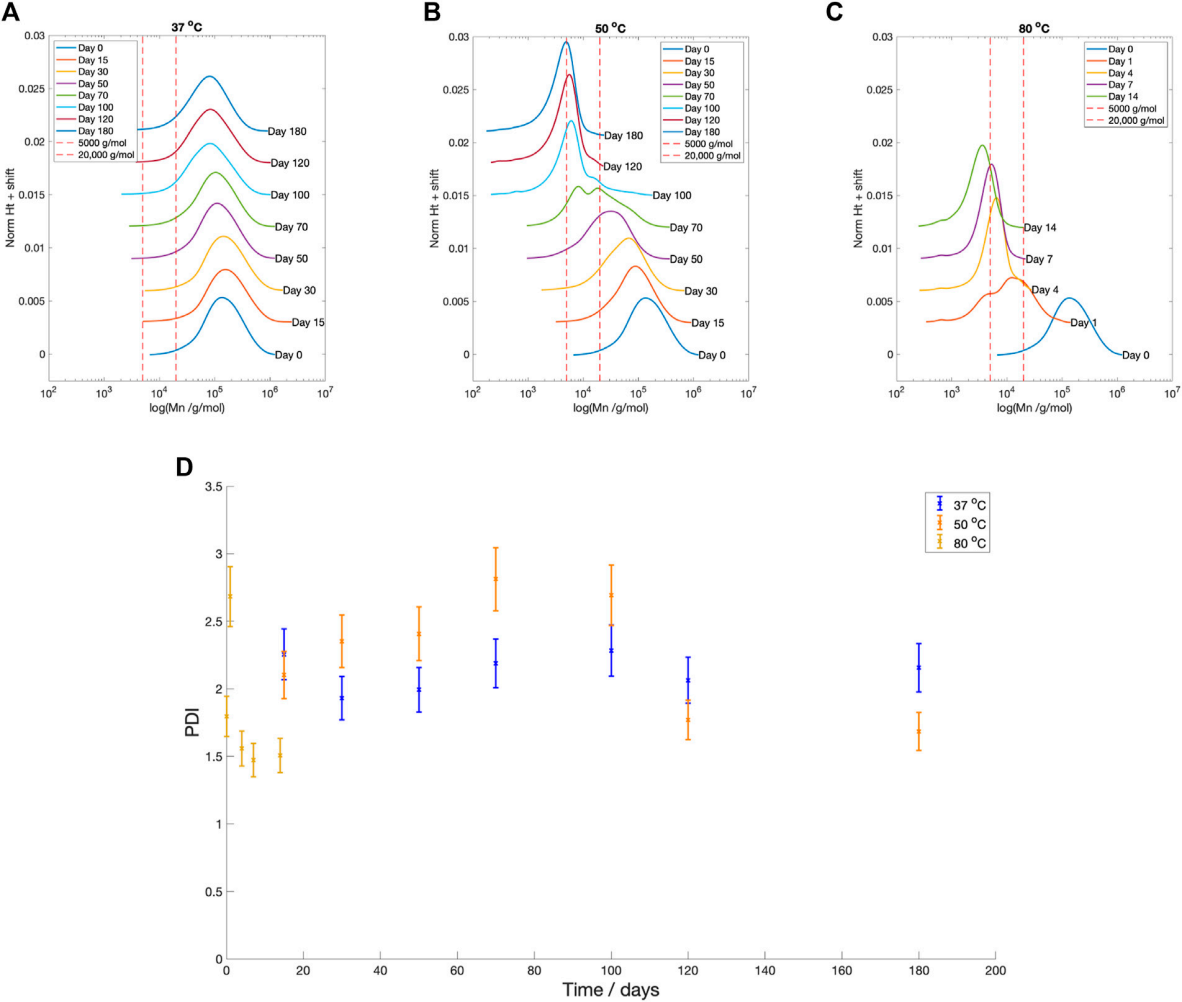
GPC profiles at degradation time for PLLA degraded in PBS at **(A)** ${37\;}^{{}^{\circ}}$ C, **(B)** ${50\;}^{{}^{\circ}}$ C, **(C)** ${80\;}^{{}^{\circ}}$ C **(D)** Polydispersity index vs. degradation time for all three temperatures ( ${37\;}^{{}^{\circ}}$ C blue, ${50\;}^{{}^{\circ}}$ C orange, ${80\;}^{{}^{\circ}}$ C yellow). By Day 22 at ${80\;}^{{}^{\circ}}$ C, not enough sample remained to run the GPC. In plots **(A–D)**, between each degradation time point a 0.003 vertical shift has added for clarity. A red vertical line at Mn values of 5,000 g/mol and 20,000 g/mol has been added.

Across all temperatures, there was a reduction in number average molecular weight (Mn) with time (Figure 5). Samples degraded at ${37\;}^{{}^{\circ}}$ C recorded the slowest rate of Mn degradation, with a 50% drop in Mn requiring approximately 75 days. At ${50\;}^{{}^{\circ}}$ C this time reduced to approximately 15 days. For degradation at ${80\;}^{{}^{\circ}}$ C, the Mn reduced over 95% within the first 24 h. The degradation rate then slowed in the following 13 days. The GPC profiles at each degradation temperature are shown in Figure 5. At ${37\;}^{{}^{\circ}}$ C, there was a gradual shift in peak position, with a single peak recorded at each degradation time. At ${50\;}^{{}^{\circ}}$ C, a bimodal peak was seen to develop at Day 70. By Day 100, a small higher molecular weight secondary peak could be seen. By Day 120, there was a return to a single peak. Finally, at ${80\;}^{{}^{\circ}}$ C, a two peak profile was seen at Day 1. By Day 4, there was only a single peak. Figure 5D plots the change in Polydispersity index (PDI) with degradation time. At ${37\;}^{{}^{\circ}}$ C all the PDI values were less than 2.50, whilst at ${50\;}^{{}^{\circ}}$ C the PDI recorded a maximum of 2.81 ± 0.23 at Day 70. At ${80\;}^{{}^{\circ}}$ C, a maximum recorded PDI value of 2.68 ± 0.22 was recorded after Day 1, before dropping to approximately 1.50 for the remaining time period.

#### 3.3.4 X-ray diffraction

**FIGURE 6:**
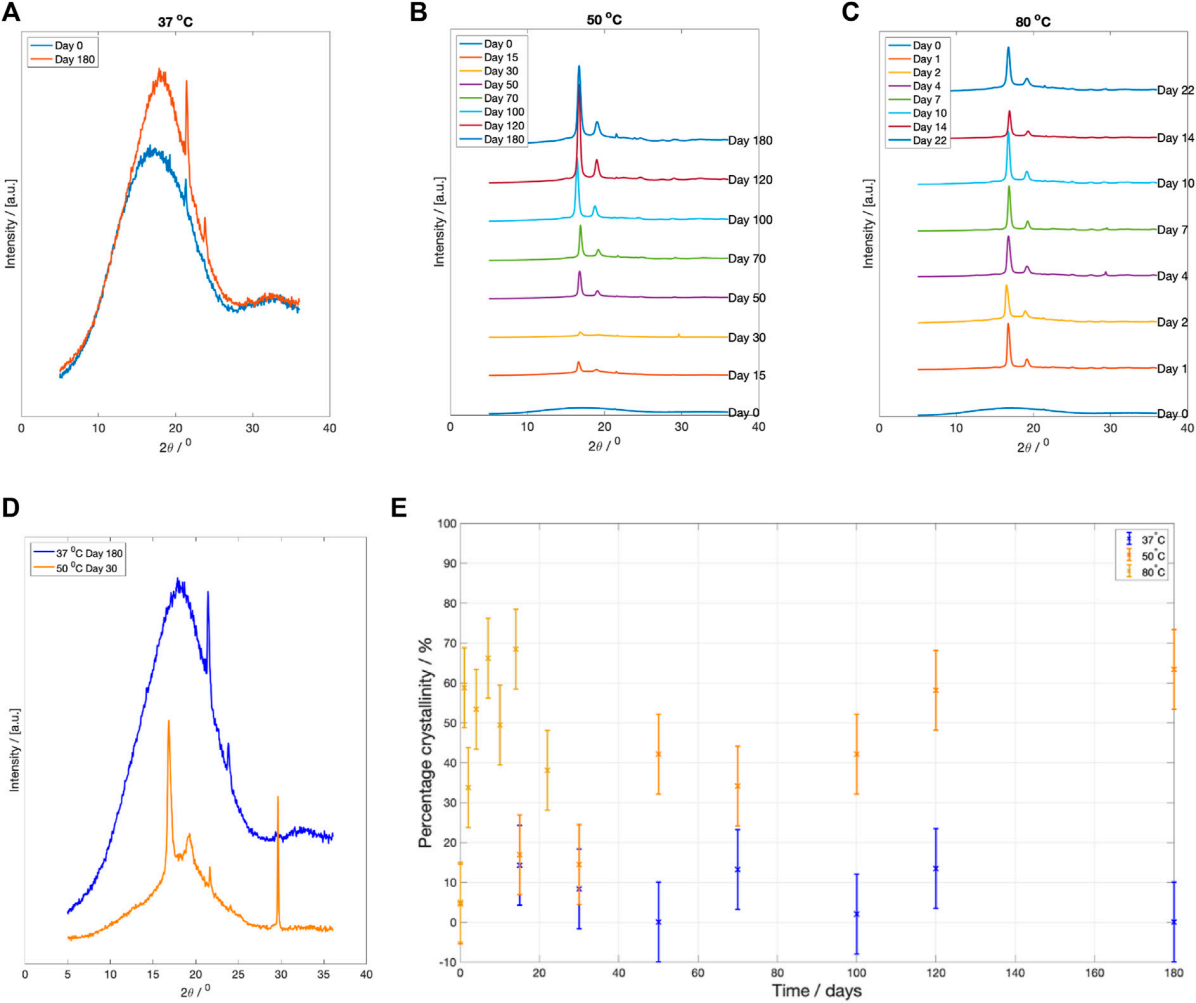
XRD scattering results at specific degradation time points after drying. **(A)** WAXS pattern for PLLA degraded at ${37\;}^{{}^{\circ}}$ C at Day 0 and 180, **(B)** WAXS patterns at each degradation time point for PLLA degraded at ${50\;}^{{}^{\circ}}$ C, and **(C)** WAXS pattern for PLLA degraded ${80\;}^{{}^{\circ}}$ C. A vertical shift, at degradation temperatures ${50\;}^{{}^{\circ}}$ C and ${80\;}^{{}^{\circ}}$ C, has been introduced between time points to make the data clearer. **(D)** Comparison of the WAXS diffraction patterns after drying for samples degraded at ${37\;}^{{}^{\circ}}$ C for 180 days vs. samples degraded for ${50\;}^{{}^{\circ}}$ C for 30 days. The recorded Mn values at this point are approximately the same. Note the difference in peak position for ${37\;}^{{}^{\circ}}$ C and ${50\;}^{{}^{\circ}}$ C fluid temperature. **(E)** Change in recorded crystallinity during hydrolysis for samples degraded at ${37\;}^{{}^{\circ}}$ C (blue), ${50\;}^{{}^{\circ}}$ C (yellow) and ${80\;}^{{}^{\circ}}$ C (yellow).

X-ray diffraction profile of samples degraded at each fluid temperature are plotted in Figure 6. After 6 months degradation at ${37\;}^{{}^{\circ}}$ C, the XRD scans still showed a broad amorphous halo (Figure 6A). There was little recorded change in the crystallinity during this period. Two peaks are located at two theta position ${21\;}^{{}^{\circ}}$ and ${24\;}^{{}^{\circ}}$ . Samples degraded at ${50\;}^{{}^{\circ}}$ C (Figure 6B) gradually developed distinct peaks at two theta positions $16^{{}^{\circ}}$ and $19^{{}^{\circ}}$ . Samples degraded at ${80\;}^{{}^{\circ}}$ C, showed a rapid development of crystalline peaks at two theta positions approximately located at $16^{{}^{\circ}}$ and $19^{{}^{\circ}}$ (Figure 6C). Figure 6D compares WAXS patterns after 180 days of degradation at ${37\;}^{{}^{\circ}}$ C and 30 days of degradation at ${50\;}^{{}^{\circ}}$ C. Samples had a similar Mn at these respective time points (45,000 g/mol at ${37\;}^{{}^{\circ}}$ C vs. 31,000 g/mol at ${50\;}^{{}^{\circ}}$ C). The two theta peak located around ${30\;}^{{}^{\circ}}$ for the sample degraded at ${50\;}^{{}^{\circ}}$ C was identified as contamination from calcium magnesium carbonate (Blanchard, 2021).

#### 3.3.5 Crystallinity

The change in crystallinity, estimated using Rietveld refinement, is plotted in Figure 6E. There was little recorded change in sample crystallinity for PLLA degraded at ${37\;}^{{}^{\circ}}$ C, with all samples remaining amorphous. At ${50\;}^{{}^{\circ}}$ C, there was a rise in crystallinity from approximately 5% to over 60% over the 180 days of degradation. At ${80\;}^{{}^{\circ}}$ C, the crystallinity increased to 66% within the first 7 days, before falling to 38% by Day 22.

#### 3.3.6 Thermal properties

**FIGURE 7:**
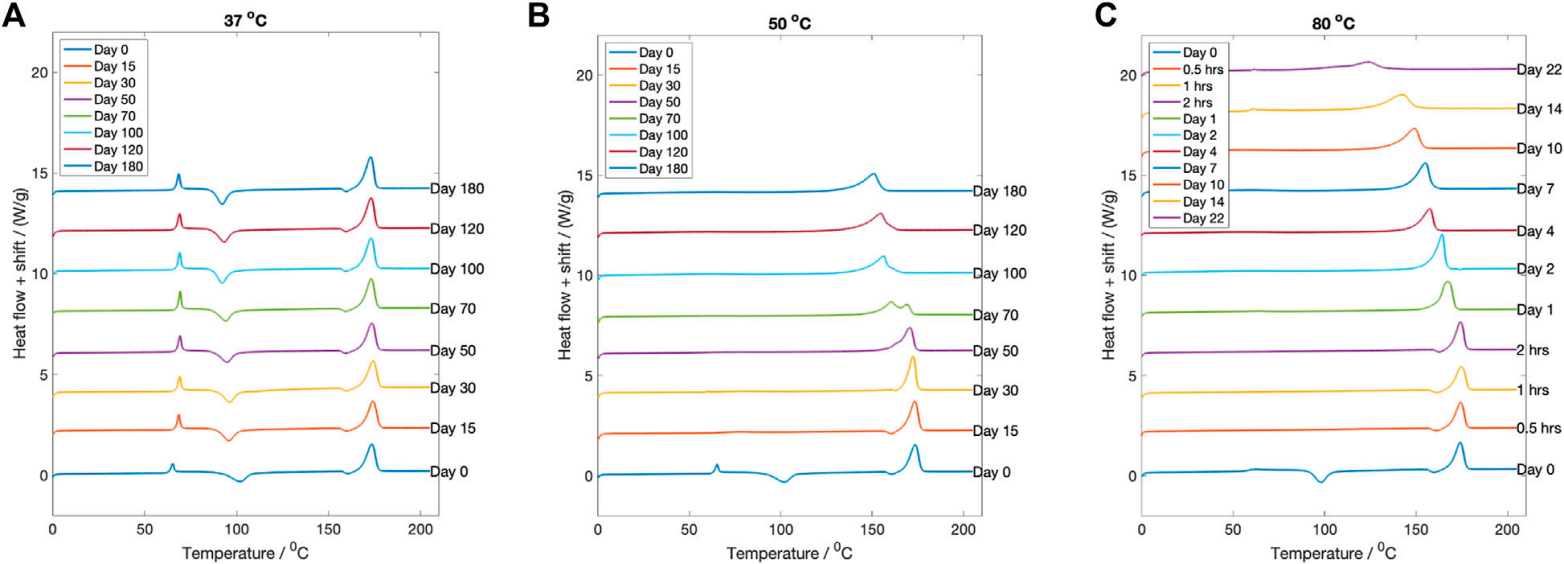
The DSC curves for each fluid temperature at every degradation time point. **(A)** ${37\;}^{{}^{\circ}}$ C, **(B)** ${50\;}^{{}^{\circ}}$ C and **(C)** ${80\;}^{{}^{\circ}}$ C. Between a degradation time point, a vertical shift of 2 W/g has been introduced for clarity.

The change in thermal properties of the dried degraded samples was investigated using DSC. For degradation at ${37\;}^{{}^{\circ}}$ C, the DSC curves are plotted in Figure 7A. A vertical shift between lines has been introduced for clarity. Both the Tg and its accompanying enthalpy relaxation peak height increased following degradation. The Tg increased from approximately 61.5 ± ${1.5\;}^{{}^{\circ}}$ C to 66.4 ± ${1.5\;}^{{}^{\circ}}$ C during the first 50 days before plateauing. At Day 180 the Tg was measured to be 68.3 ± ${1.5\;}^{{}^{\circ}}$ C. The size and shape of the Tg peak suggests an enthalpy relaxation behaviour has occurred. Simultaneously, there was a fall in cold crystallisation temperature from 100.3 ± ${1.5\;}^{{}^{\circ}}$ C to 92.3 ± ${1.5\;}^{{}^{\circ}}$ C over the degradation time period. No change in the final melting temperature in the DSC was recorded across the 180 day period.

Conversely, for samples degraded at ${50\;}^{{}^{\circ}}$ C (Figure 7B) the glass transition and cold crystallisation were not observed after degradation for any time period measured. After 50 days of degradation, the melting temperature reduced to 170.7 ± ${1.5\;}^{{}^{\circ}}$ C, and then further reduced to 152.2 ± ${1.5\;}^{{}^{\circ}}$ C by Day 180.

Samples degraded at ${80\;}^{{}^{\circ}}$ C (Figure 7C) recorded a flattening to their Tg and cold crystallisation, during the initial 24 h period. After this, the recorded melting temperature gradually reduced. After 10 days the melting temperature was recorded as 149.1 ± ${1.5\;}^{{}^{\circ}}$ C, and by Day 22 it was recorded as 124.2 ± ${1.5\;}^{{}^{\circ}}$ C.

#### 3.3.7 Mechanical properties

**FIGURE 8:**
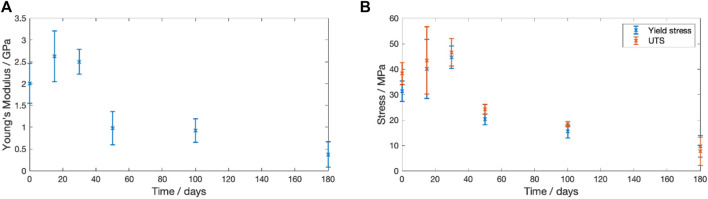
Change in the recorded mechanical properties for PLLA degraded in PBS at ${37\;}^{{}^{\circ}}$ C over a 180 day period. **(A)** Young’s modulus **(B)** Yield stress (blue) and **(C)** Ultimate tensile stress (orange) vs. degradation time.

The change in Young’s modulus, yield stress and ultimate tensile strength (UTS) for dried samples degraded at ${37\;}^{{}^{\circ}}$ C is shown in Figure 8. It was seen that all three parameters initially increased during the first 30 days of degradation. After this, all three parameters then steadily fell throughout the remaining 150 days. The Young’s modulus declined from a starting value of 2.00 ± 0.46 GPa to 0.98 ± 0.39 GPa at Day 50. At Day 180 there was a further decline in Young’s modulus to 0.37 ± 0.29 GPa. The UTS shifted from a peak value of 46.54 ± 5.43 MPa to a final value 7.68 ± 5.52 MPa. The samples exhibited increasingly brittle behaviour with the difference between yield stress and UTS tending to zero with prolonged degradation.

## 4 Discussion

### 4.1 Three-stage degradation model

**FIGURE 9:**
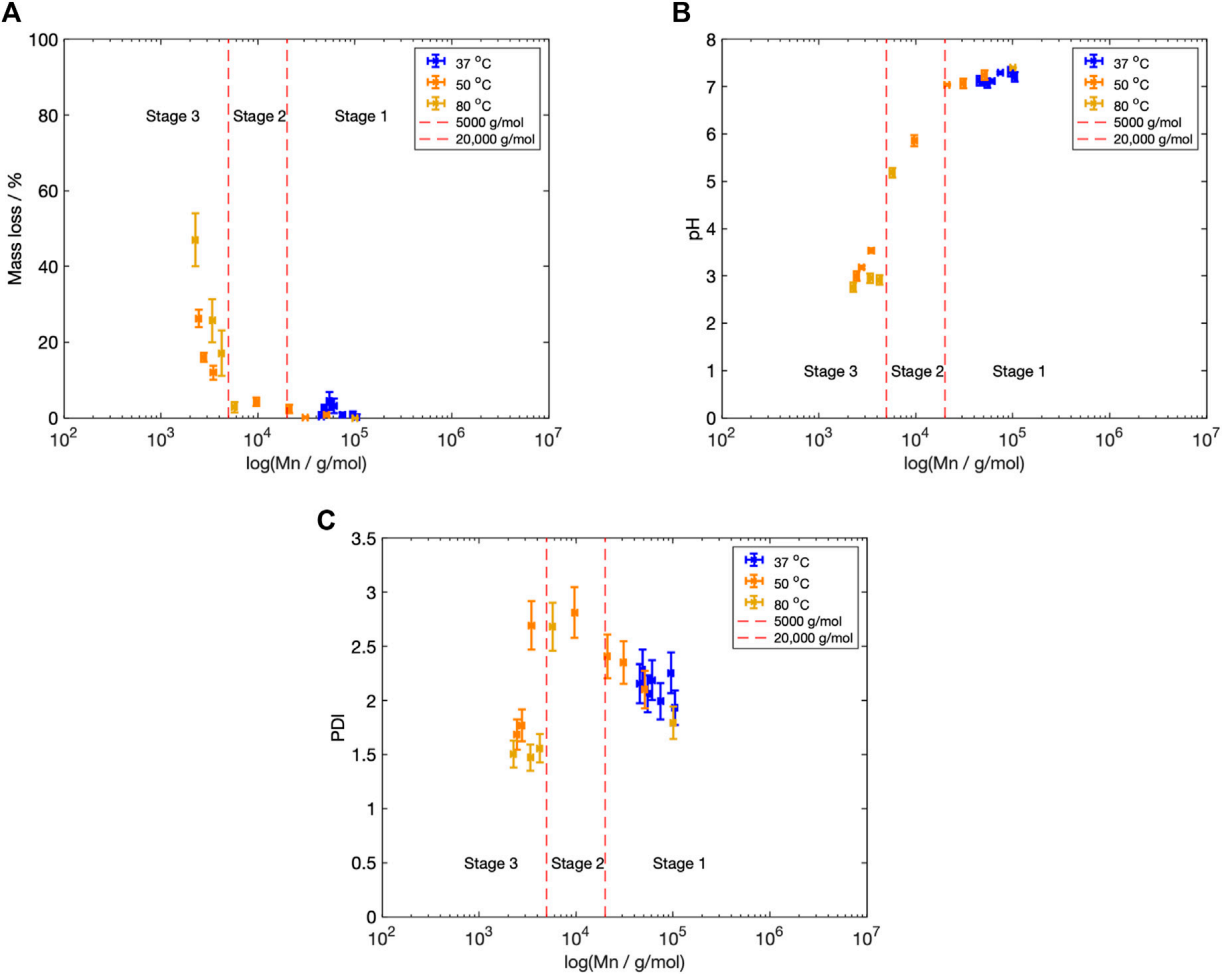
**(A)** Mn vs. mass loss across all three fluid temperatures, ${37\;}^{{}^{\circ}}$ C (blue), ${50\;}^{{}^{\circ}}$ C (orange) and ${80\;}^{{}^{\circ}}$ C (yellow) **(B)** Mn vs. pH at each fluid temperature. **(C)** Polydispersity index (PDI) vs. Mn at each fluid temperatures. The three degradation stages have been marked, along with the approximate stage boundaries.

When the data from the pH and mass loss experiments were combined with the molecular weight data, across all fluid temperatures, a three-stage degradation process could be identified (Figure 9). Previous studies have subdivided the degradation of poly( α -hydroxy esters) into a number of stages. However, the exact number and precise stage classification is still debated within the literature (Li, 1999; Middleton and Tipton, 2000; Alexy and Levi, 2013; Limsukon et al., 2023). By breaking down the degradation into distinct stages, we can gain a better understanding of the overall degradation process, and more accurately model the behaviour of the polymer at each stage.

The Mn boundaries between each stage are approximate; however, the overall categorisation was based on trends across the entire data set and previous literature. The boundary between Stage 1 and 2 was determined by the increase in the PDI from 2.40 to greater than 2.60 (Figure 5D), as well as the reduction in fluid pH (Figure 4), once the Mn fell below 20,000 g/mol (Figure 5). Weir et al. also reported the onset of mass loss for 0.8 mm annealed PLLA once the sample molecular weight fell below 20,000 (Weir et al., 2004a). The boundary between Stage 2 and 3 was based upon the drop in the PDI from 2.60 to below 2.00 (Figure 5D), alongside the increase in mass loss (Figure 4) that occurred when Mn shifted to less than 5,000 g/mol (Figure 5). Grizzi et al. similarly reported the onset of significant mass loss once the molecular weight was less than 7,000 g/mol, for 0.3 mm thick compression moulded amorphous poly(D-L lactic acid) (Grizzi et al., 1995; Sevim and Pan, 2018).

**Stage 1: Mn** > **20,000 g/mol** During Stage 1 there was a reduction in Mn from its 100,598 g/mol starting value to approximately 20,000 g/mol (Figure 5). There was little change to the sample mass or fluid pH throughout this stage (Figure 4). This suggests that the sample was undergoing bulk erosion rather than surface erosion. The diffusion of degradation products out of a bulk eroding structure is slow (Middleton and Tipton, 2000). Conversely, for a sample undergoing surface degradation, a linear erosion profile would be expected (von Burkersroda et al., 2002).

**Stage 2: 5,000** < **Mn** < **20,000 g/mol** Once the molecular weight was less than 20,000 g/mol, mass loss and pH changes were measured (Figure 4), marking the onset of Stage 2. This reduction in sample mass and fluid pH indicates that the degraded short-chain monomers and oligomers were small enough to diffuse out of the microstructure. The release of these carboxylic acid end groups into the fluid would then reduce the fluid pH. As the fluid pH approached and eventually surpassed the pKa point of lactic acid (3.86), the pH plateaued, most likely because the lactic acid began to exist in its protonated form (Eyal and Canari, 1995).

**Stage 3: Mn** < **5,000 g/mol** Once the molecular weight reached 5,000 g/mol, samples were determined to have crystallinity above of 50% (Figure 6E), marking the onset of Stage 3. The crystalline regions within the microstructure are commonly termed ‘crystalline residues’ (Tsuji, 2002; Tsuji et al., 2004; Auras et al., 2010). The GPC profile contained a single peak, with the poldispersity index less than its starting value (Figure 5D). This suggests that the microstructure was relatively homogeneous, containing several crystalline residues. Fischer et al. previously determined that single crystals of PLLA degrade from the outer edges (Fischer et al., 1973). The samples studied here appear to be following a similar degradation pathway, with a steady rate of polymer erosion and mass loss (Figure 4).

### 4.2 Molecular weight changes in Stage 1 and the effect on mechanical properties

#### 4.2.1 Equation to represent molecular weight evolution with time in Stage 1

Two models have been proposed to represent the reduction in Mn, prior to the onset of mass loss (Stage 1). Anderson et al. suggested a statistical approach with the molecular weight following an inverse relationship with degradation time, t (Eq. (2)):

$$\frac{1}{M_{N}}=\frac{1}{M_{N0}}+k_{1}t$$ (2)

where $M_{N}$ is the number average molecular weight, $M_{N0}$ starting molecular weight, $k_{1}$ rate constant and t the degradation time.

The model assumes that no autocatalysis has occurred. Pitt and Gu developed an alternative model to account for the autocatalysis of the carboxylic acid end groups generated during the degradation (Pitt et al., 1981). Their model predicted an exponential relationship with degradation time, t (Eq. (3)):

$$M_{N}=M_{N0}e^{-k_{2}t}$$ (3)

Neither model can be applied to samples degraded at ${80\;}^{{}^{\circ}}$ C, where mass loss occurred within 24 h.

Table 1 shows that at ${37\;}^{{}^{\circ}}$ C, both the degradation models of Anderson et al. and Pitt or Gu fit well. The overall degradation rate was slow, and thus the influence of autocatalysis within the 6 month period was minimal. For degradation at ${50\;}^{{}^{\circ}}$ C, there was marginally better fitting with the autocatalytic model. At both fluid temperatures, values are compared with previous literature, either Weir et al. at ${37\;}^{{}^{\circ}}$ C, or Weir et al. and Chausse et al. at ${50\;}^{{}^{\circ}}$ C (Weir et al., 2004a; Chausse et al., 2023). Weir et al. samples were 0.8 mm annealed PLLA degraded in PBS. Chausse et al. samples were 3D printed 120.9 μ m thick samples, annealed for 12 h prior to degradation in PBS. At both degradation temperatures, there was found to be good agreement between calculated rate constants in the present work and previous literature.

**TABLE 1:** Rate constants and accompanying $R^{2}$ for the uncatalysed and autocatalysed models for samples degraded at ${37\;}^{{}^{\circ}}$ C and ${50\;}^{{}^{\circ}}$ C. Table also predicts the time required for degraded samples to reach a Mn of 20,000 g/mol for both degradation models. This Mn marks the onset of Stage 2.

Author and References	Temperature/°C	Uncatalysed, $k_{1}$		Time to reach Stage 2/days	Catalysed, $k_{2}$		Time to reach Stage 2/days
		Rate constant/days	$R^{2}$		Rate constant/days	$R^{2}$	
Present work	37	7.58* $10^{-8}$	0.85	528	0.0050	0.81	323
Weir et al. (Weir et al., 2004a)	37	9* $10^{-8}$	0.63	-	0.0052	0.85	-
Present work	50	7.64* $10^{-7}$	0.99	48	0.0311	0.96	53
Weir et al. (Weir et al., 2004a)	50	3* $10^{-7}$	0.90	-	0.0196	0.96	-
Chausse et al. (Chausse et al., 2023)	50	2.64* $10^{-7}$	0.88	-	0.0188	0.99	-

Using the degradation models developed by Anderson et al. and Pitt and Gu respectively, the time to reach a molecular weight of 20,000 g/mol was determined (Table 1). This marks the boundary between Stage 1 and 2 and the approximate onset of mass loss and pH changes (Figure 9).

#### 4.2.2 The dependency of mechanical properties on molecular weight in Stage 1

**FIGURE 10:**
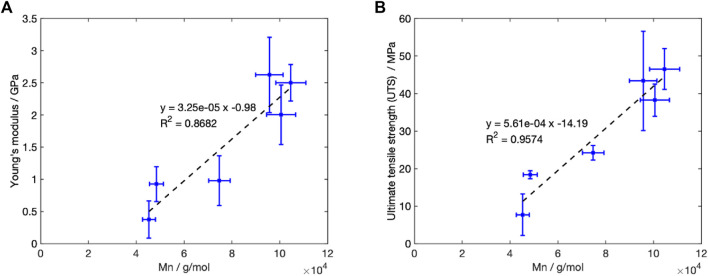
Change in the recorded mechanical properties with Mn for degradation at ${37\;}^{{}^{\circ}}$ C **(A)** Young’s modulus vs. Mn. A linear trend line has been added with an $R^{2}$ value of 0.87. **(B)** Ultimate tensile strength (UTS) vs. Mn. An $R^{2}$ value of 0.96 was recorded.

The reduction in Mn during Stage 1, was accompanied by a change in mechanical properties at ${37\;}^{{}^{\circ}}$ C. Figure 10 plots the change in Young’s modulus and the UTS with the Mn. A linear fit for both parameters ( $R^{2}$ fitting of 0.87 and 0.96 for Young’s modulus and UTS respectively) was determined, agreeing with Weir et al.’s reported behaviour for annealed PLLA (Weir et al., 2004a).

### 4.3 Mass and molecular weight changes in Stage 3

#### 4.3.1 Equation to represent mass change with time in Stage 3

In Stage 3, oligomer and short-chain monomers were predicted to move via diffusion from the sample into the surrounding fluid. This diffusion was assumed to obey Fick’s diffusion laws. As such, Stefan’s approximation predicting the change in mass over time was fitted (Eq. (4)) (Moliner et al., 2020).

$$\frac{M_{t}}{M_{s}}=\frac{4}{l}{\left(\frac{Dt}{\pi }\right)}^{\frac{1}{2}}$$ (4)

Where $M_{t}$ is the mass at time t, $M_{s}$ saturation mass (amount of fluid initially absorbed by the structure), D the diffusion coefficient, l the diffusion length and t the degradation time.

**FIGURE 11:**
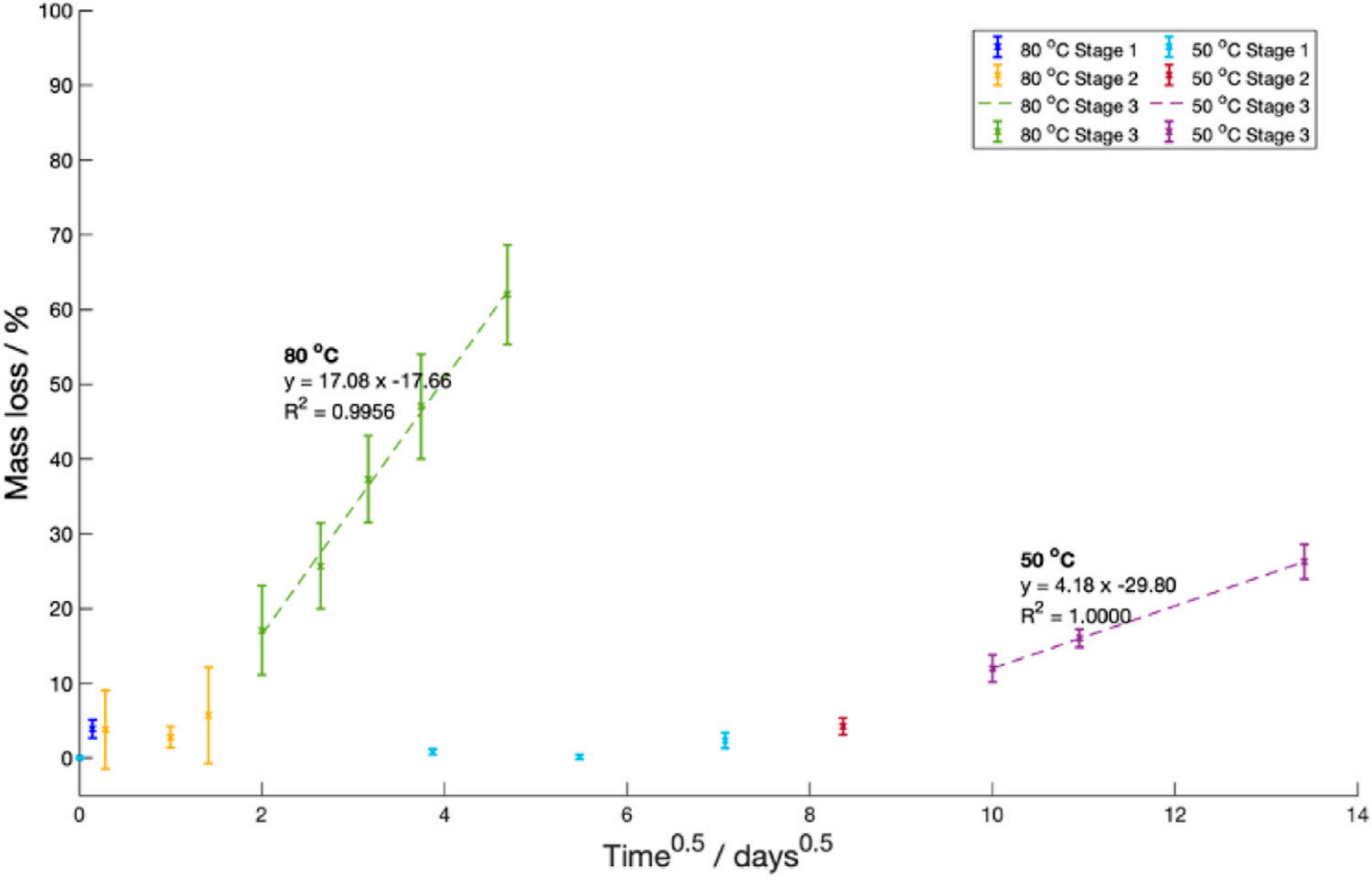
Mass loss against the square route of the degradation time at ${50\;}^{{}^{\circ}}$ C (light blue, red and purple) and ${80\;}^{{}^{\circ}}$ C (navy, yellow and green). For both temperatures, Stages 1 and 2 (prior to the onset of mass loss) are shown in different colours for clarity.

A constant saturation mass, diffusion length and coefficient are assumed in Eq. (4). $M_{t}$ can then be replaced with the percentage mass loss at time t. There was good fitting between Stefan’s equation and the data collected at both ${50\;}^{{}^{\circ}}$ C and ${80\;}^{{}^{\circ}}$ C, both $R^{2}$ values were 0.99, once Stage 3 was reached (Figure 11).

#### 4.3.2 Evolution of molecular weight with time in Stage 3

Tsuji et al. previously found that the molecular weight of crystalline residues decreased with a linear time dependency (Tsuji and Ikarashi, 2004a; Tsuji and Ikarashi, 2004b). They manufactured their starting crystalline residues by degrading as-cast PLLA 100 μ m films at ${97\;}^{{}^{\circ}}$ C for 40 h in PBS. Their crystalline residue starting molecular weight was 10,200 g/mol. For the data collected in the present work, we found that once the recorded Mn was below 5,000 g/mol, the reduction in molecular weight could similarly be modelled with a linear time dependency. Table 2 lists the calculated experimental rate constants, comparing them to Tsuji et al. values. For samples degraded at ${80\;}^{{}^{\circ}}$ C, Mn values from Day 4 onwards are used. For samples degraded at ${50\;}^{{}^{\circ}}$ C, Mn values from Day 100 onwards are used.

**TABLE 2:** Linear rate constant during Stage 3 degradation. For the present work data, k is calculated from Day 100 onwards at ${50\;}^{{}^{\circ}}$ C and Day 4 onwards at ${80\;}^{{}^{\circ}}$ C.

Author and References	Temperature/°C	k/g/mol/day
Present work	50	11
Present work	80	190
Tsuji et al. (Tsuji and Ikarashi, 2004b)	50	28
Tsuji et al. (Tsuji and Ikarashi, 2004b)	70	86
Tsuji et al. (Tsuji and Ikarashi, 2004b)	97	710

There was good matching between the calculated rate constant at ${80\;}^{{}^{\circ}}$ C for the present work and Tsuji et al. value. However, the rate constant calculated at ${50\;}^{{}^{\circ}}$ C for the present work was smaller than Tsuji et al.’s reported value. The PDI value at Day 100 for samples degraded at ${50\;}^{{}^{\circ}}$ C was 2.69 ± 0.22, larger than Tsuji et al.’s starting value (1.23). Secondly, the GPC curve contained a small secondary peak (Figure 5B), which could indicate the presence of some preferentially degraded amorphous material within the microstructure. This is consistent with the XRD scattering results in Figure 6E which recorded the presence of amorphous material within the microstructure. The presence of amorphous material could explain the difference in rate constant between the two data sets.

### 4.4 Overall degradation pathway as a function of temperature

In Figures 5B,C, the development of multi-modal peaks during Stage 2 at ${50\;}^{{}^{\circ}}$ C and ${80\;}^{{}^{\circ}}$ C fluid temperatures, suggests that some parts of the microstructure were being selectively degraded. Previous studies have suggested that multi-modal GPC peaks occur when the core of the sample is degrading faster relative to the surface (Li et al., 1990; Grizzi et al., 1995). The good fitting between the Pitt and Gu’s autocatalytic hydrolysis model and the current Mn data set, might suggest that this has occurred within this studies degraded samples. However, this explanation cannot explain the observed increase in crystallinity at ${50\;}^{{}^{\circ}}$ C and ${80\;}^{{}^{\circ}}$ C (Figure 6E). Alternatively, some studies have suggested that the formation of a double peak within the Mn curves occurs due to the preferential hydrolysis of the amorphous regions (Fischer et al., 1973; Li et al., 1990; Pistner et al., 1993; Tsuji and Ikada, 1997; Tsuji and Ikada, 1998; Tsuji et al., 2000; Chye et al., 2005). The selective hydrolysis of the amorphous regions would explain the recorded increase in crystallinity seen at ${50\;}^{{}^{\circ}}$ C and ${80\;}^{{}^{\circ}}$ C. However, across all fluid temperatures, there were only marginal increases to the measured PDI values (Figure 5D). The recorded PDI values in the present study are very similar in size to those reported by Weir et al. for 0.8 mm annealed PLLA, degraded in PBS at ${37\;}^{{}^{\circ}}$ C (Weir et al., 2004b). They suggested their samples were degrading homogeneously, with little surface to core, or autocatalytic degradation occurring. It may not be possible for us to determine exactly which explanation and degradation pathway occurred, and it is likely that multiple pathways were concurrently active.

In Figure 9, the overall degradation was divided into three-stages. Samples degraded at ${37\;}^{{}^{\circ}}$ C did not advance beyond Stage 1 during the studied time period. The suggested degradation behaviour in Stages 2 and 3 is only based on the data sample degradation at ${50\;}^{{}^{\circ}}$ C and ${80\;}^{{}^{\circ}}$ C. At ${37\;}^{{}^{\circ}}$ C, the sample Mn remained greater than 20,000 g/mol throughout the 180 days degradation period.

#### 4.4.1 Degradation at ${80\;}^{{}^{\circ}}$ C

Above Tg, changes to polymer orientation or crystallinnity can occur. For samples degraded at ${80\;}^{{}^{\circ}}$ C the increase in crystallinity was expected, with the recorded observations fitting this behaviour. A maximum crystallinity of 66% was recorded after 7 days (Figure 6E). With further hydrolysis, the sample crystallinity reduced, possibly due to disruption of the crystal structure during crystalline residue break-up (von Recum et al., 1995). Visually the PLLA turned white during the first 24 h and with prolonged degradation the material formed a fine powder (Figure 3C). The reduction in the amount of connecting amorphous material present produced a structure which fell apart under its own weight. The melting point reduced from approximately ${172\;}^{{}^{\circ}}$ C to ${124\;}^{{}^{\circ}}$ C over the 22 days as Mn reduced (Figure 7C). The reduction in final melting temperature is consistent with a gradual reduction in crystal thickness (Tsuji and Ikarashi, 2004b).

#### 4.4.2 Degradation at ${37\;}^{{}^{\circ}}$ C

Conversely, for samples degraded at ${37\;}^{{}^{\circ}}$ C, the polymer was below its Tg and there was little change in polymer crystallinity during degradation (Figure 6E). The results in the present work agree with Tsuji and Suzuyoshi who reported that for amorphous PLLA, less than 50 μ m thick, no increase in crystallinity was observed over a 70 day period (Tsuji and Suzuyoshi, 2002). The two theta peaks located at ${21\;}^{{}^{\circ}}$ and ${24\;}^{{}^{\circ}}$ (Figure 6A) are not believed to have arisen from scattering from a PLLA crystal structure, since peaks in this two theta position have not been reported elsewhere for any PLLA crystal structure (Ru et al., 2016; Lotz et al., 2017). It seems likely that the scattering was due to sample contamination. The peaks were identified to be from Polyethylene (PE) (see the Supplementary Material for more information). The DSC results (Figure 7A) indicated that upon reheating, the PLLA underwent an enthalpy relaxation. This relaxation is indicative of a physical ageing process and suggests that glass densification may have occurred (Vyavahare et al., 2014). This densification might explain the rise in Tg from 6 ${1.5\;}^{{}^{\circ}}$ C to ${69\;}^{{}^{\circ}}$ C (Pan et al., 2008). Conversely, the increase in polymer chain mobility in the hydrated state might explain the gradual shift in the cold crystallisation temperature from ${97\;}^{{}^{\circ}}$ C to ${92\;}^{{}^{\circ}}$ C (Vyavahare et al., 2014). The physical ageing within the amorphous regions at ${37\;}^{{}^{\circ}}$ C agrees with previous reported results (Tsuji, 2002; Vyavahare et al., 2014). The GPC profile showed a gradual reduction in Mn, and no increase in PDI (Figure 5A). This suggests that either no preferential degradation of the amorphous regions or autocatalysis occurred. The findings at ${37\;}^{{}^{\circ}}$ C (no increase in crystallinity and no evidence of autocatalysis) contrast with some previous results. Li et al. found that amorphous PLLA degraded at ${37\;}^{{}^{\circ}}$ C had an approximately linear increase in crystallinity over a 2-year period (Li et al., 1990). The crystallinity rose from 0% to 17% during the initial 31-week period. Li et al. starting Mn was slightly smaller than the degraded samples used within this study, approximately 72,000 g/mol compared with 100,598 g/mol respectively. Importantly however, their samples were 2 mm thick, and an autocatalysed core clearly developed. The larger sample thickness, may be crucial to explain both the autocatalysis and their recorded increase in crystallinity during degradation.

During the 180 days degradation there were significant changes to the mechanical properties. Figure 8 shows that during the first 30 days of degradation there was an increase in the Young’s Modulus and UTS. However, the PLLA transitioned from elastic-plastic behaviour to brittle behaviour. The recorded behaviour matches that reported by Pan et al. who found that physical ageing of 380–400 μ m thick amorphous PLLA at ${40\;}^{{}^{\circ}}$ C for 465 h, increased the tensile modulus and yield strength, whilst simultaneously reducing the fracture strain (Pan et al., 2007). Cui et al. suggested that the development of large internal stresses during the ageing process can lead to brittle failure, with the interplay between the decreasing free volume and the development of internal stresses determining the final mechanical and thermal properties (Cui et al., 2020). With prolonged degradation, the Young’s modulus of samples in the present study, dropped. Chain cleavage has been shown to reduce the Van der Waals forces between polymer chains, which in turn can reduce the Young’s modulus (Ding et al., 2012).

#### 4.4.3 Degradation at ${50\;}^{{}^{\circ}}$ C

For degradation occurring at ${50\;}^{{}^{\circ}}$ C, PLLA was below its dry starting glass transition ( ${60\;}^{{}^{\circ}}$ C). Accordingly, there should be little change to the sample crystallinity. However, Figure 6E showed that sample crystallinity increased to over 60% during the 6 month period. To explain this result the effect of hydration on the Tg (Figure 2B) must be considered. Fluid immersion reduced the Tg, and as a result, the chains underwent structural rearrangement and crystallised. A similar magnitude of Tg reduction was reported by Siemann for Poly-D,L-lactic acid (P(DL)LA) (Siemann, 1985). The shift in Tg was a hydration effect, not a degradation effect, as the Tg rebounded to start value after vacuum drying (Figure 2B). Furthermore, the reduction in Tg to at or below the fluid temperature might explain why crystalliation was happening faster than enthalpy relaxation for samples degraded at ${50\;}^{{}^{\circ}}$ C.

This reduction in Tg when immersed in fluid, meant the samples followed the behaviour recorded at ${80\;}^{{}^{\circ}}$ C. Visually, there was a significant transition to a more opaque colour, and after 100 days the samples were no longer intact (Figure 3B). The DSC curves indicated that, relative to the starting microstructure, after 50 days little amorphous material was present (Figure 7B). Any remaining amorphous material after this time appeared to be rigid amorphous, with the chains tightly bound to the crystalline regions. The removal of mobile amorphous regions, and in particular the tie chains between the crystalline domains, may explain the sample break-up. With additional degradation time, a subsequent reduction in sample crystallinity would be expected, due to the disruption of the crystal structure during crystalline residue break-up.

It is important to consider whether the Tg of samples degraded at ${37\;}^{{}^{\circ}}$ C would eventually reach that of the surrounding fluid. Passerini and Craig previously reported that the lowering of Tg in fluid obeyed the Gordon-Taylor relationship (Passerini and Craig, 2001). Using their suggested relationship, to lower the Tg of the PLLA used in the present study from its starting value of 6 ${1.5\;}^{{}^{\circ}}$ C to ${37\;}^{{}^{\circ}}$ C, a water content (saturation mass) in excess of 6% would be required. PLLA is a hydrophobic polymer, and when Weir et al. conducted a degradation experiment for 66 weeks at ${37\;}^{{}^{\circ}}$ C, a maximum water content of 2.5% was found (Weir et al., 2004b). By this time the samples started to lose mass as short chain monomers and oligomers diffused out. This suggests that the sample Tg would not lower below ${37\;}^{{}^{\circ}}$ C, and that the samples are not expected to crystallise when immersed in fluid at this temperature even after a prolonged time.

#### 4.4.4 Extrapolation from ${50\;}^{{}^{\circ}}$ C to ${37\;}^{{}^{\circ}}$ C

This study has shown a clear difference between the degradation behaviour at different fluid temperatures. When degraded samples with a similar Mn at different temperatures are compared, significant differences between the material microstructures were found. After 30 days degradation at ${50\;}^{{}^{\circ}}$ C the Mn was approximately 31,000 g/mol. Similarly, after 6 months of degradation at ${37\;}^{{}^{\circ}}$ C, the Mn was approximately 45,000 g/mol. However, the XRD scans at these two time points are markedly different (Figure 6D). By Day 30 at ${50\;}^{{}^{\circ}}$ C, several crystalline peaks have begun to emerge. However, at ${37\;}^{{}^{\circ}}$ C, the PLLA microstructure recorded a broad amorphous halo. The divergence between the microstructures would be expected to widen with prolonged degradation time. It appears that degrading amorphous PLLA at elevated temperatures, regardless of whether the temperature is above or below the starting Tg, changes the degradation pathway.

To determine whether extrapolation from ${50\;}^{{}^{\circ}}$ C to ${37\;}^{{}^{\circ}}$ C is valid, irrespective of the change in degradation pathway, the Arrhenius equation (Eq. (5)) can be used to extrapolate from ${50\;}^{{}^{\circ}}$ C to ${37\;}^{{}^{\circ}}$ C (Weir et al., 2004a; Chausse et al., 2023). The calculated results can be compared with the collected experimental data for the present work. The rate constants determined at ${50\;}^{{}^{\circ}}$ C, were on the same order of magnitude as Weir et al. (Weir et al., 2004a) (Table 1). When Weir et al. applied the Arrhenius equation to their data set they calculated the activation energy required for Mn loss to be $E_{a}$ to be 100.5 kJ/mol. Applying this value to the present work, for both the autocatalysed model and the uncatalysed degradation models, a calculated rate constant for ${37\;}^{{}^{\circ}}$ C was generated (Table 3). The table also lists the percentage difference between the calculated and experimental rate constant. Table 4 lists the time in days to reach a Mn value of 20,000 g/mol, the onset of mass loss. Again the percentage difference between the calculated and experimental based values are given.

$$k=Ae^{-\frac{E_{a}}{RT}}$$ (5)

where k is the rate constant, A is a constant, $E_{a}$ the activation energy, R the universal gas constant, and T the temperature in Kelvin.

**TABLE 3:** Extrapolation using the Arrhenius equation and the experimentally measured ${50\;}^{{}^{\circ}}$ C data set, a rate constant at ${37\;}^{{}^{\circ}}$ C can be calculated. The percentage difference between the calculated and experimental rate constant is shown.

Degradation model	Calculated rate constant	Experimental rate constant	Percentage difference
Uncatalysed	1.59* $10^{-7}$	7.58* $10^{-8}$	109
Autocatalysed	0.0065	0.0050	30

**TABLE 4:** The time to reach Stage 2, at ${37\;}^{{}^{\circ}}$ C, for the calculated rate constants (determined using the Arrhenius equation) and the experimental rate constant calculated within this study. The percentage difference between the two values is listed.

Degradation model	Days to reach 20,000 g/mol		Percentage difference
	Calculated rate constant	Experimental rate constant	
Uncatalysed	252	528	52
Autocatalysed	250	323	23

The values in Tables 3 and 4 show that at best, there is a 30% difference between the calculated rate constant and experimental rate constant, which leads to a 23% difference in time to reach the onset of mass loss. Based on this data, it appears that Arrhenius temperature extrapolation is not a suitable method for predicting the lifespan of amorphous PLLA at ${37\;}^{{}^{\circ}}$ C.

The results gathered from this study suggest that 3D printed PLLA degrading at ${37\;}^{{}^{\circ}}$ C undergoes bulk degradation. However, the results have not found clear evidence for the formation of an autocatalytic region within the 100 μ m thick amorphous PLLA fibres. Furthermore, there was no recorded increase in sample crystallinity during degradation at ${37\;}^{{}^{\circ}}$ C. This is contrast to the recorded behaviour at ${50\;}^{{}^{\circ}}$ C and ${80\;}^{{}^{\circ}}$ C. At elevated fluid temperatures, it appears there is a change in degradation pathway. This change effects the validity of temperature extrapolation, and renders temperature extrapolation an unsuitable method to accurately predict the functional lifespan of amorphous PLLA degraded at ${37\;}^{{}^{\circ}}$ C. Amorphous samples should instead be tested at body temperature. The degradation models developed by Anderson et al. and Pitt and Gu can successfully predict the change Mn prior to the onset of mass loss. The provisional data from the present work suggests that late stage mass loss can be modelled using a diffusion-based approach.

## 5 Conclusion

This work investigated the degradation pathway of 100 μ m thick 3D printed amorphous PLLA, degrading in PBS over 6 months. The results collected in the present work suggest that the PLLA fibres underwent homogeneous bulk degradation. However, there were significant microstructural changes dependent upon the fluid temperature. At ${37\;}^{{}^{\circ}}$ C, the PLLA microstructure remained amorphous, but there was a densification process during the degradation period. Conversely, at ${50\;}^{{}^{\circ}}$ C and ${80\;}^{{}^{\circ}}$ C, XRD revealed that the PLLA crystallised, suggesting the PLLA underwent an alternative degradation pathway. A hydration study revealed that immersion of PLLA in PBS, lowered the Tg, thereby enabling crystallisation at temperatures below its dry starting Tg. Due to the difference in degradation pathway between the PLLA degraded at ${37\;}^{{}^{\circ}}$ C, and the PLLA degraded at ${50\;}^{{}^{\circ}}$ C or ${80\;}^{{}^{\circ}}$ C, we suggest that using accelerated degradation testing, at elevated temperatures, to predict the *in-vivo* lifespan is not valid. Instead, for future *in-vivo* applications, samples should be tested at ${37\;}^{{}^{\circ}}$ C. These findings are important when considering the degradation behaviour of future biomedical devices containing amorphous PLLA fibres.

## Data Availability

The raw data supporting the conclusions of this article will be made available by the authors, without undue reservation.
