# Supplementary material for: Accelerated degradation testing impacts the degradation processes in 3D printed amorphous PLLA
Source: Front Bioeng Biotechnol. 2024 Jul 5;12:1419654. doi: 10.3389/fbioe.2024.1419654 (PMC11257899; doi:10.3389/fbioe.2024.1419654)
Supplement: Supplementary file 1 [file DataSheet1.pdf]

Accelerated degradation testing impacts the degradation processes in 3D printed amorphous PLLA

Luke P Malone [1,*], **Serena M Best** [1] **and Ruth E Cameron** [1]

[1] *Cambridge Centre for Medical Materials (CCMM), Department of Materials Science and Metallurgy, University of Cambridge, United Kingdom*

Correspondence*:
Luke Malone
lpm39@cam.ac.uk

SUPPLEMENTARY MATERIAL

The presence of two theta peaks 21 ^{0} and 24 ^{0} in the X-ray scattering data collected for samples degraded at 37 ^{0}C, are not believed to have arisen due to PLLA. A Rietveld refinement on TOPAS based on the work of Takahashi identified the peaks as PE contamination (1). Figure 1 plots the Rietveld fitting, and the original experimental data for comparison, for a sample degraded at 37 ^{0}C for 15 days. Smaller contamination peaks were identified in other degraded samples, as well as the Day 0 samples. We therefore suspect that the initial material contained PE contamination.

FIGURE AND CAPTION

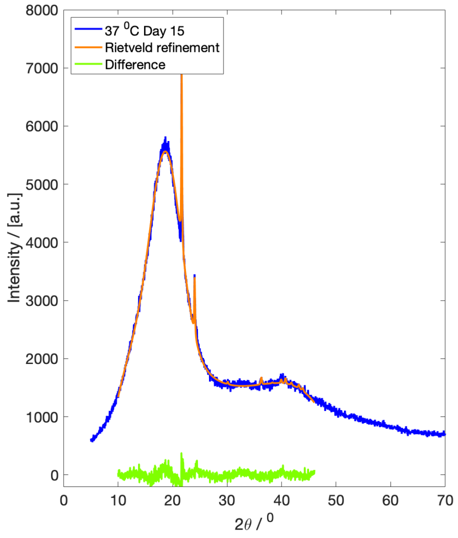

Figure 1. Plotting the WAXS data for PLLA degraded at 37 ^{0}C for 15 days (blue), Rietveld fitting (orange), and the difference between the two lines (green). Rietveld refinement based on the work of Takahashi (1).

REFERENCES

1 .Takahashi Y. Neutron Structure Analysis of Polyethylene-d4. *Macromolecules* **31** (1998) 3868–3871. doi:10.1021/ma9706790.
